# Arabidopsis Protein Phosphatase PIA1 Impairs Plant Drought Tolerance by Serving as a Common Negative Regulator in ABA Signaling Pathway

**DOI:** 10.3390/plants12142716

**Published:** 2023-07-21

**Authors:** Yan Huang, Rongqian Yang, Huiling Luo, Yuan Yuan, Zhihong Diao, Junhao Li, Shihe Gong, Guozhi Yu, Huipeng Yao, Huaiyu Zhang, Yi Cai

**Affiliations:** College of Life Sciences, Sichuan Agricultural University, Ya’an 625000, China

**Keywords:** *Arabidopsis*, ABA signaling, PP2C, PIA1, negative regulator

## Abstract

Reversible phosphorylation of proteins is a ubiquitous regulatory mechanism in vivo that can respond to external changes, and plays an extremely important role in cell signal transduction. Protein phosphatase 2C is the largest protein phosphatase family in higher plants. Recently, it has been found that some clade A members can negatively regulate ABA signaling pathways. However, the functions of several subgroups of *Arabidopsis* PP2C other than clade A have not been reported, and whether other members of the PP2C family also participate in the regulation of ABA signaling pathways remains to be studied. In this study, based on the previous screening and identification work of PP2C involved in the ABA pathway, the clade F member PIA1 encoding a gene of the *PP2C* family, which was down-regulated after ABA treatment during the screening, was selected as the target. Overexpression of *PIA1* significantly down-regulated the expression of ABA marker gene *RD29A* in *Arabidopsis* protoplasts, and ABA-responsive elements have been found in the cis-regulatory elements of *PIA1* by promoter analysis. When compared to Col-0, transgenic plants overexpressing *PIA1* were less sensitive to ABA, whereas *pia1* showed the opposite trait in seed germination, root growth, and stomatal opening experiments. Under drought stress, SOD, POD, CAT, and APX activities of *PIA1* overexpression lines were lower than Col-0 and *pia1*, while the content of H_2_O_2_ was higher, leading to its lowest survival rate in test plants, which were consistent with the significant inhibition of the expression of ABA-dependent stress-responsive genes *RD29B*, *ABI5*, *ABF3*, and *ABF4* in the *PIA1* transgenic background after ABA treatment. Using yeast two-hybrid and luciferase complementation assays, PIA1 was found to interact with multiple ABA key signaling elements, including 2 RCARs and 6 SnRK2s. Our results indicate that *PIA1* may reduce plant drought tolerance by functioning as a common negative regulator involved in ABA signaling pathway.

## 1. Introduction

Plants, on the one hand, are constantly challenged by various biological or abiotic stresses throughout their lifetime, while on the other hand, they continuously develop regulatory mechanisms that can respond to these stresses in order to survive [1]. Reversible protein phosphorylation is a ubiquitous regulatory mechanism in organisms that can promptly respond to external changes. Previous studies have revealed that it is not only an important part of the cell signal transduction pathway [2], but also plays a key role in plant responses to stress [3,4,5,6,7,8]. The completion of reversible protein phosphorylation depends mainly on protein kinases (PKs) and protein phosphatases (PPs), which act as “switches” in the signaling pathway to regulate protein activity [9,10,11]. Unlike that of PKs, which have been well studied, the function of most PPs and the link between protein dephosphorylation and signaling pathways are currently unknown due to functional redundancy among the phosphatase-encoding genes.

Based on the substrate specificity, PPs can be divided into protein tyrosine phosphatase (PTP), serine (Ser)/threonine (Thr)- specific protein phosphatase (PPP), and metal-dependent protein phosphatase (PPM) [12], among which type 2C protein phosphatase (PP2C) Ser/Thr protein phosphatase is the largest branch of the protein phosphatase family [13]. PP2C exists widely in organisms in the form of monomeric enzymes, and its activity is closely related to the metal ions such as Mg^2+^, Ca^2+^, Zn^2+^, and Mn^2+^ [14], but is not inhibited by known phosphatase inhibitors such as okadaic acid [15]. Studies have shown that PP2C can regulate cell growth and development through dephosphorylation and regulate changes in related genes in vivo through different signal transductions to resist stress [16]. In addition, compared to other organisms, PP2C in plants is the most abundant and widely present in a variety of species, including *Arabidopsis* [13], rice [13], maize [17], cabbage [18], and cotton [19]. The abundant number of PP2C indicates that the signal transduction mechanism in different tissues has a certain diversity, which makes it very likely to be involved in regulating plant growth and development and responding to stress through different signal pathways. At present, PP2C is usually associated with abscisic acid (ABA) in plant abiotic stress responses, which is considered to be an early discovered regulatory factor in ABA signaling pathway [7].

As an endogenous plant hormone, ABA has a marked effect on multiple physiological processes throughout the plant life cycle, including seed dormancy and germination, root growth, stomatal guard cell expansion, leaf senescence, and fruit ripening [20,21,22,23,24], and is also involved in plant responses to abiotic stress, such as osmotic stress, salt stress, drought stress, low-temperature stress, and oxidative stress [25,26,27,28,29,30,31,32]. Plants respond to ABA signaling pathways by recognizing ABA in plant cells and converting ABA stimuli into a series of physiological and biochemical responses through signal transduction [33]. In the classical ABA signaling model, PP2C physically interacts with SnRK2 (SNF1-related protein kinase 2) and thus inhibits SnRK2 kinase activity in the absence of ABA. In the presence of ABA, ABA molecules bind to the PYR/PYL/RCAR (Pyrabactin resistance/PYR-like protein/regulatory component of ABA receptor) to change the receptor conformation, and further combine with PP2C to form the ABA-PYR/PYL/RCAR-PP2C ternary complex to inhibit the activity of PP2C, thus causing the release of SnRK2. Subsequently, SnRK2 phosphorylates and activates the downstream targets in response to external stress [34,35,36,37].

According to the structural characteristics of *Arabidopsis PP2C* genes [38], they can be further divided into 10 groups (A-J), excluding 6 unclustered groups. At present, the research on the A clade members of PP2C is the most extensive, and a large number of studies have shown that the A clade members of PP2C are mainly involved in ABA signal regulation [39,40,41]. For example, ABI1, ABI2, and HAB1 are members of the PP2C A clade identified in Arabidopsis to negatively regulate the ABA signaling pathway [42,43,44], while AaPP2C1 in *Artemisia annua* had a similar function [45]. Maize ZmPP2C-A2, ZmPP2C-A6, and ZmPP2C-A10 and tomato SlPP2C3 can reduce plant drought tolerance by acting as negative regulators of ABA signals [46,47,48]. The relationship between the function of PP2C A clade members and ABA signaling has been well established, but whether other members of the PP2C family are also involved in the regulation of ABA signaling is unclear. In our previous study, we screened the non-A member genes of the *Arabidopsis PP2C* family that may be involved in the regulation of the ABA signaling pathway by using the protoplast transient expression system. Among the multiple non-A member *PP2Cs* identified in the screen, overexpression of F member encoding gene *PIA1* can significantly down-regulate the expression level of ABA pathway marker genes in Arabidopsis protoplasts, but the specific mechanism involved remains to be elucidated.

In this study, on the basis of verification that overexpression of *PIA1* can significantly inhibit the expression of ABA pathway reporter *pRD29A* using the Arabidopsis protoplast transient expression system and the presence of ABA-responsive elements in cis-regulatory elements of *PIA1* through promoter analysis, the stable overexpression and functional deletion lines of *PIA1* were further constructed, screened, and identified. Using them as materials, the biological function of *PIA1* was analyzed by testing seed germination, root length, stomatal opening, and ABA-dependent stress response gene expression with/without ABA treatment, as well as by detecting the activity of various antioxidant enzymes and hydrogen peroxide levels under drought stress. In addition, the interactions between PP2C and RCARs or SnRK2s were analyzed by Y2H and LCA to determine their interaction networks under abiotic stress. Our data clearly indicate that PIA1 may reduce drought tolerance in plants by acting as a common negative regulator in ABA signaling pathways.

## 2. Results

### 2.1. PIA1 with ABA-Responsive Elements Down-Regulated pRD29A in Arabidopsis Protoplasts under ABA Treatment

**Figure 1:**
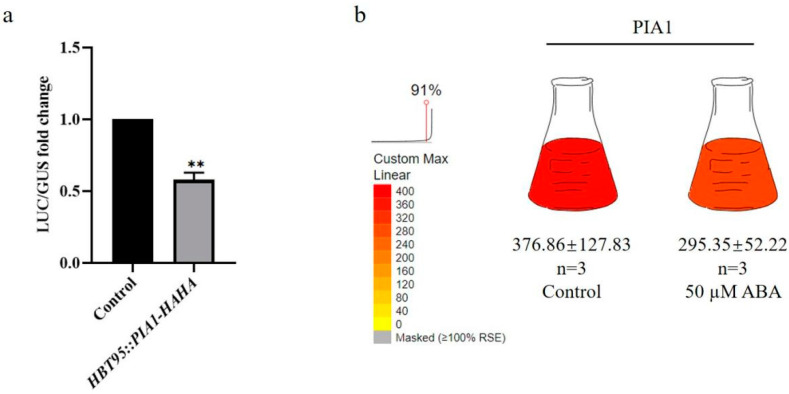
Effect of *PIA1* on ABA signaling pathway. (**a**) Effects of transient overexpression of *PIA1* on ABA pathway reporter gene *RD29A* in *Arabidopsis* protoplasts. ** *p* < 0.01. (**b**) The transcription level of *Arabidopsis PIA1*. The data is from Böhmer et al.; the pictures were downloaded and modified from ePlant.

To confirm the accuracy of our preliminary protoplast screening results, *p35S::PIA1*, *pRD29A::LUC*, and *p35S::GUS* were transformed together into *Arabidopsis* protoplasts under ABA treatment, and the effect of transient *PIA1* expression on *pRD29A* levels was detected. Using the transformed *p35S::HAHA* empty vector as a control, the expression of *pRD29A* was found to be significantly inhibited by *PIA1* (Figure 1a), which was consistent with the previous screening results.

To determine whether the protein phosphatase PIA1 is directly involved in the abiotic stress response, the promoter sequence upstream of its start codon was analyzed using the PlantCARE database, and several abiotic stress responsive cis-elements (ABRE, TC rich repeats, CGTCA-motif, TGACG-motif, MBS, TCA-element) were identified. Moreover, *PIA1* has one or more additional stress-responsive cis-elements in its promoter region, suggesting its potential involvement in various stress responses (Table 1). In addition, Böhmer et al. [49] found that the *PIA1* transcript level was down-regulated in the *Arabidopsis* Col-0 cell line T87 after a 50 µM ABA treatment (Figure 1b), which was similar to our findings that transient-expression *PIA1* in *Arabidopsis* protoplasts down-regulated ABA-dependent *RD29A* expression. To further identify the biological function of *PIA1*, we constructed stable OE *PIA1s* and identified a *pia1* mutant from Arashare. Transgenic lines and mutants were verified by PCR and Western blot analysis (Figure S1).

### 2.2. PIA1 in Arabidopsis Affected ABA Sensitivity

**Figure 2:**
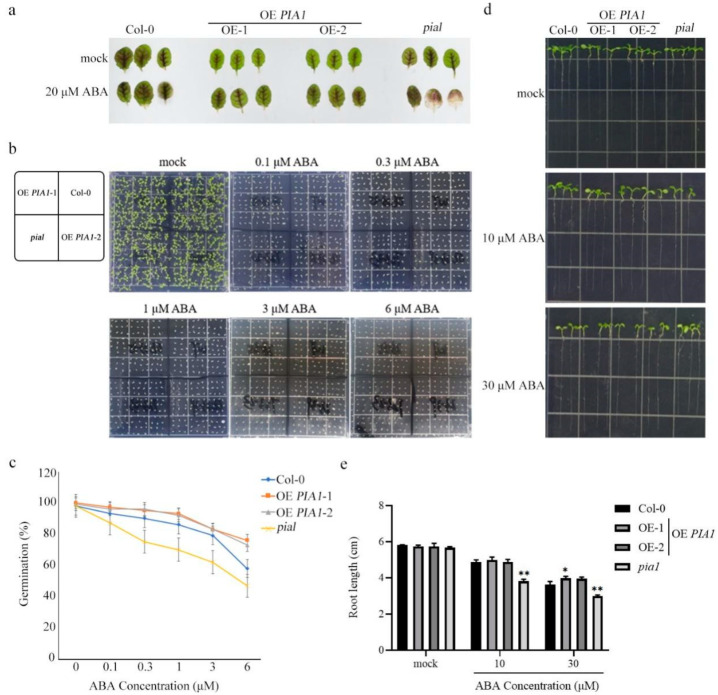
The phenotype of leaves, seed germination, and root length. (**a**) The phenotype of leaves of Col-0, OE *PIA1*, and *pia1* mutants. (**b**) The seed germination of Col-0, OE *PIA1*, and *pia1* mutants. 100 seeds were germinated and grown on ½ MS plate containing ABA, and photographed on the 11th day. (**c**) Statistics of the seed germination. (**d**) The root length of Col-0, OE *PIA1*, and *pia1* mutants. The seeds were germinated and grown on 1/2 MS plates for 7 days, then transferred to the plate containing ABA for 4 days, and then we took photos and count the root length. (**e**) Statistics of the root length. Data are means ± SE of three replicates. Significances of differences are indicated as * *p* < 0.05, ** *p* < 0.01.

To investigate whether *PIA1* is related to plant response to ABA, the leaves of 4-week-old Col-0, OE *PIA1*, and *pia1* were immersed in water and water supplemented with 20 μM ABA. Compared with Col-0, the leaves of OE *PIA1* showed no significant difference, whereas the leaves of the mutant *pia1* showed a whitening phenotype associated with ABA sensitivity (Figure 2a). Since seed germination is closely related to ABA response, statistical analyses of seed germination rates in Col-0, OE *PIA1,* and *pia1* under different concentrations of ABA were performed. The results showed that the germination rate of *PIA1* overexpression lines was significantly higher than that of Col-0 on 1, 3, and 6 μM ABA plates, whereas the germination rate of *pia1* was significantly lower than that of Col-0 (Figure 2b,c). Root length was also used as an indicator of ABA response to detect the effect of *PIA1* on plant response to ABA. Consistent with the above results, compared with Col-0, although the overexpression of *PIA1* had no significant effect on root length under ABA treatment, the loss of *PIA1* significantly inhibited root elongation (Figure 2d,e).

### 2.3. PIA1-Regulated ABA-Mediated Stomatal Opening and Plant Drought Tolerance

**Figure 3:**
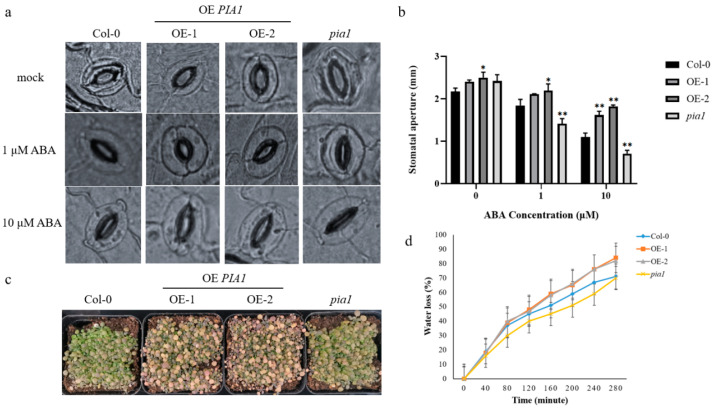
The stomatal aperture comparison, soil drought test, and water loss rate analysis. (**a**) The stomatal aperture analyses of Col-0, OE *PIA1*, and *pia1* mutants. (**b**) Statistics of the stomatal aperture. (**c**) Two-week-old plants of Col-0, OE *PIA1*, and *pia1* mutants grown in short-light conditions were not irrigated for 15 days. (**d**) Analysis of water loss rate from detached leaves. Data are means ± SE of three replicates. Significances of differences are indicated as * *p* < 0.05, ** *p* < 0.01.

It is known that plants can regulate stomatal opening and reduce transpiration by synthesizing endogenous ABA to improve their drought tolerance under stress [50]. Therefore, we further verified the effect of *PIA1* on ABA-mediated stomatal opening, as well as plant drought tolerance under stress. Under normal conditions, the stomatal aperture of *PIA1* transgenic seedlings was slightly larger than that of Col-0 and *pia1*. However, after ABA treatment, stomatal apertures of all tested seedlings decreased, with the largest reduction in the mutant, followed by Col-0, and transgenic plants was the least (Figure 3a,b). Similarly, *PIA1* transgenic seedlings exhibited a more sensitive phenotype than Col-0 and *pia1* in the soil drought stress test, with more leaves turning yellow and dying from dehydration (Figure 3c). Further water loss rate measurements confirmed the phenotype observed above (Figure 3d).

### 2.4. PIA1 Regulated the Activities of Antioxidant Enzymes and H_2_O_2_ Content under Drought Stress or Altered Expressions of Drought-Responsive Genes under ABA Treatment

**Figure 4:**
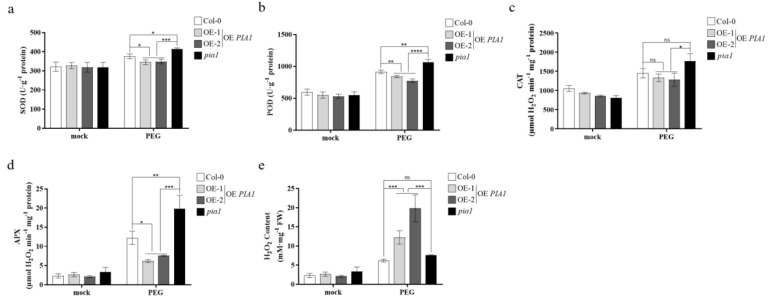
Antioxidants and oxidant enzymes determination under drought condition. (**a**) SOD activity. (**b**) POD activity. (**c**) CAT activity. (**d**) APX activity. (**e**) H_2_O_2_ content. Significances of differences are indicated as ns indicates no significant, * *p* < 0.05, ** *p* < 0.01, *** *p* < 0.001, **** *p* < 0.0001.

The activities of SOD, POD, CAT, and APX and H_2_O_2_ content were measured to preliminarily analyze the physiological mechanism of *PIA1* involved in plant responses to drought stress. As shown in Figure 4a–e, the decreased drought tolerance caused by overexpression of *PIA1* under drought stress might be related to the significantly increased H_2_O_2_ content under a transgenic background, which was caused by the significantly decreased activities of SOD, POD, and APX, rather than CAT. 

**Figure 5:**
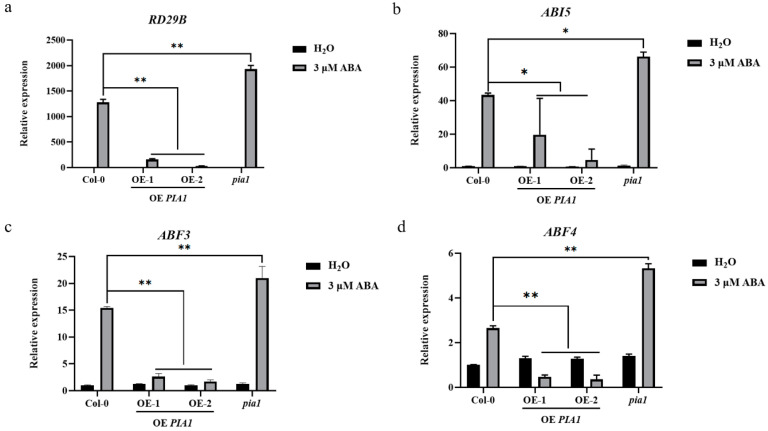
Expression patterns of relevant genes in Col-0, OE *PIA1*, and *pia1* seedlings. (**a**) *RD29B*. (**b**) *ABI5*. (**c**) *ABF3*. (**d**) *ABF4*. Significances of differences are indicated as * *p* < 0.05, ** *p* < 0.01.

To identify molecular events involved in *PIA1*-mediated ABA signaling, expressions of several ABA-responsive genes, including *RD29B*, *ABI5*, *ABF3*, and *ABF4* were investigated. The results showed that the expression levels of all detected genes were significantly down-regulated in *PIA1* transgenic plants under ABA treatment and were lower than that of Col-0; results were the opposite in a *pia1* mutant background (Figure 5a–d). 

### 2.5. PIA1 Interacted with Several Elements in the Classical ABA Signaling Model 

**Figure 6:**
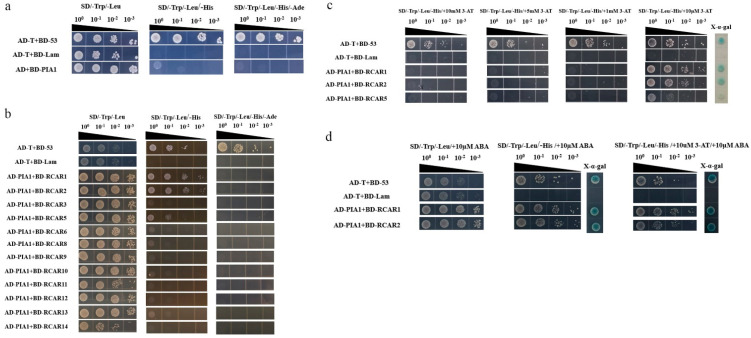
PIA1 interacts with 2 RCARs. (**a**) BD-PIA1 has no self-activation. (**b**–**d**) PIA1 interacts with RCAR1, RCAR2, but not with RCAR3, RCAR5, RCAR6, RCAR8, RCAR9, RCAR10, RCAR11, RCAR12, RCAR13, RCAR14.

Studies have found that ABA receptor proteins and SnRK2 family protein kinases participate in ABA signaling pathway and are regulated by group A PP2C [51,52,53,54,55]. In order to identify the target proteins of PIA1 involved in ABA signaling pathway, it was determined, by Y2H, whether the interactions were between PIA1 and 12 RCARs or 10 SnRK2s. On the premise of confirming no self-activation of BD-PIA1 (Figure 6a), we first examined the interactions between PIA1 and RCARs. It was found that PIA1 possibly interacted with RCAR1, RCAR2, and RCAR5 in the absence of exogenous ABA (Figure 6b). In order to verify the authenticity of the interactions, 3-AT was added to the SD/-Trp/-Leu/-His plates to exclude the influence of the Ade of yeast-competent cells, and X- α- Gal staining was performed, revealing that PIA1 interacted with RCAR1 and RCAR2 rather than RCAR5 (Figure 6c). Those interactions were then further confirmed not to be altered by the presence of ABA (Figure 6d).

**Figure 7:**
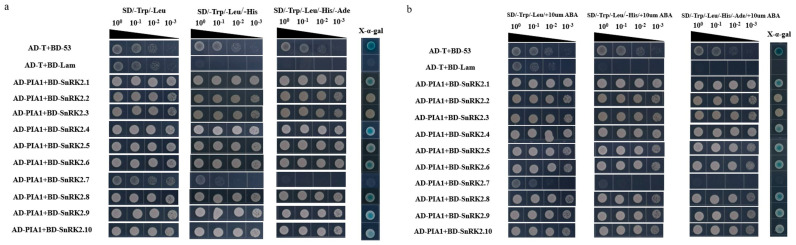
PIA1 interacts with 6 SnRK2s. (**a**,**b**) PIA1 interacts with SnRK2.1, SnRK2.4, SnRK2.5, SnRK2.6, SnRK2.8, and SnRK2.9, but not with SnRK2.2, SnRK2.3, SnRK2.7, or SnRK2.10.

**Figure 8:**
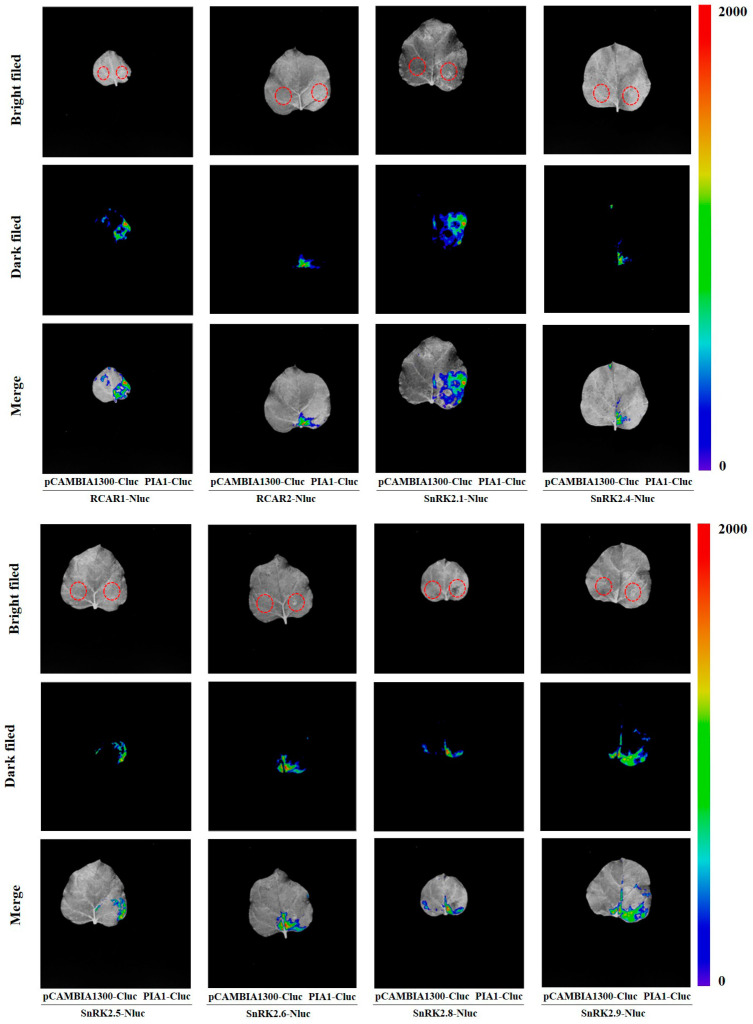
PIA1 interacts with 2 RCARs and 6 SnRK2s.

Next, we examined the interactions between PIA1 and SnRK2s, and found that PIA1 interacted with six of the SnRK2s (SnRK2.1, SnRK2.4, SnRK2.5, SnRK2.6, SnRK2.8, and SnRK2.9) regardless of the absence/presence of ABA (Figure 7). Finally, we further demonstrated that PIAI interacted with several elements in the classical ABA signal transduction model, including 2 RCARs and 6 SnRK2s, by LCA (Figure 8, Figure S2).

## 3. Discussion

As a recognized stress hormone, ABA signaling pathway that response to abiotic stress in plants has been well elucidated, and key components involved have been identified, including ABA receptors, protein phosphatases, protein kinases, transcription factors, and other related elements [33]. On this basis, a classic ABA-PYR/PYL/RCAR-PP2C-SnRK2 signal transduction model has been established in *Arabidopsis* [34,35,36,37]. In this model, PP2C is the most diverse due to its unique physical and chemical properties, structural characteristics, and large quantities. At present, the studies on stress related to ABA regulation is mainly focused on class A PP2Cs [40,56,57,58,59], but whether other subfamily members were involved remains to be clarified. In previous studies, we used the protoplast transient expression system to systematically screen non-A class member genes of the Arabidopsis *PP2C* family that may be involved in ABA signaling regulation, and found several potential targets, including *PIA1*. In this study, on the basis of the verification of the preliminary transient screening result (Figure 1a), combined with promoter analysis and Böhmer et al.’s findings (Figure 1b), it was revealed that the cis-regulatory elements of the *PIA1* promoter include ABRE and the ABA-responsive elements [60,61], and we speculated that *PIA1* might play a negative regulatory role in ABA signaling.

It’s known that ABA plays an important role in the physiological processes of plant growth and development [62,63,64], and is also related to plant responses to abiotic stress [65], such as inhibiting seed germination and root growth and promoting stomatal closure to reduce water loss during drought. To test our hypothesis, we examined the effects of the overexpression and loss of function of *PIA1* on ABA-induced seed dormancy (Figure 2b,c), root growth inhibition (Figure 2d,e), stomatal closure (Figure 3a,b), and drought stress gene expression (Figure 5), as well as on antioxidant enzyme activities and hydrogen peroxide content (Figure 4) under drought stress. It was found that overexpression of *PIA1* reduced plant susceptibility to ABA, whereas *pial* exhibited increased susceptibility to ABA. Notably, the overexpression of *PIA1* had no significant effect on root growth inhibition (Figure 2d,e), suggesting that *PIA1* may not be a major negative regulatory gene involved in ABA regulation of the root growth pathway, but *pial* showed a different phenotype from Col-0, which might be due to the dose effects of hormones. Meanwhile, we also found that endogenous ABA-mediated drought tolerance was inhibited in the *PIA1* transgenic background and enhanced in *pia1* mutants under natural drought conditions (Figure 3c,d). So far, we have found that *PIA1* can negatively regulate ABA signaling by down-regulating the positive regulatory factors encoding genes, including *RD29B*, *ABI5*, *ABF3,* and *ABF4* in the ABA pathway [66,67], and inhibiting multiple antioxidant enzyme activities, which leads to an increase in intracellular hydrogen peroxide content and causes oxidative damage, thus increasing the sensitivity of plants to drought stress.

Since PPs function is mainly achieved through the dephosphorylation of substrates, understanding PP2C substrates is of great significance for exploring their mechanism of action. The established ABA-PYRs/PYLs/RCARs-PP2Cs-SnRK2s signaling model in *Arabidopsis* provides a basis for the screening of targets of PIA1 negatively regulating ABA signaling pathway. Using Y2H and LCA techniques, it was found that PIA1 interacted with both 2 RCARs and 6 SnRK2s, and their interactions were independent of ABA (Figure 6, Figure 7 and Figure 8, Figure S2). Because of the identification of multiple targets, the regulatory effect of PIA1 on ABA appears to be more general than specific. The subsequent detection of dephosphorylation of PIA1 and identification of key dephosphorylation sites are indispensable to elucidate its mechanism. Even more, future work will identify more PP2Cs that regulate the ABA signaling pathway, and completing their functional analysis will help to enrich the classical ABA signaling model.

## 4. Materials and Methods

### 4.1. Plant Materials and Growth Conditions

*Arabidopsis* plants used include: the *Arabidopsis thaliana* ecotype Colombia (Col-0), *AT2G20630* overexpression lines (OE *PIA1*), and a *pia1* mutant (*SALK_105978C*) from AraShare. *Nicotiana benthamiana* (*N. benthamiana*) plants used for the luciferase complementation assay (LCA) experiment were planted in nutrient soil and grown at 24 °C with 16 h of light for 5 weeks. *Arabidopsis* plants used for stomatal closure, soil drought test, and water loss rate analysis experiments were planted in nutrient soil and grown at 24 °C with 16 h of light for 4 weeks. For seed germination, root length, and qPCR experiments, seeds were sterilized with 75% alcohol and planted on 1/2 Murashige Skoog (MS) medium (1/2 MS medium containing 3% sucrose and 0.7% agar). After 2 days of vernalization at 4 °C, seeds were cultured vertically for 10 days at 24 °C with 16 h of light.

### 4.2. Vector Construction

The primers for constructing plant overexpression vectors, yeast two-hybrid (Y2H) vectors, and luciferase complementation vectors were designed according to the instructions of a one-step cloning kit (Vazyme, Nanjing, China). Table S1 summarizes primer and vector information. 

### 4.3. Arabidopsis Protoplasts Transient Expression Assay

Transient expression in *Arabidopsis* protoplasts was performed as described previously [68]. *HBT-PIA1-HAHA*, *pRD29A::LUC*, and *p35S::GUS* were transformed together into *Arabidopsis* protoplasts under ABA treatment and cultured for 12 h before detection of *pRD29A::LUC* and GUS activity. *HBT-HAHA* was used as a control.

### 4.4. Promoter Cis-Acting Element Analysis

We used the PlantCARE (http://www.dna.affrc.go.jp/htdocs/PLACE/) (accessed on 28 May 2022) online prediction purpose gene promoter of all kinds of cis elements.

### 4.5. Phenotyping of Transgenic Arabidopsis

3 leaves of Col-0 mutants and transgenic plants grown for 4 weeks under short sunlight conditions were placed in an aqueous solution containing ABA at a final concentration of 20 µM or without ABA, respectively, and incubated in white light for 3 days and then photographed. The seedlings of Col-0, mutant, and transgenic plants grown for 3 weeks under short-day conditions were subjected to drought treatment without watering, and photographs were taken when obvious phenotypic differences appeared. 5 leaves of plants grown for 4 weeks under short-day conditions were cut and weighed at different time intervals to calculate the rate of water loss.

### 4.6. Seed Germination and Root Length

Seed germination and seedling growth were observed daily for 11 days after sowing on 1/2 MS plate with ABA. The root lengths of plants were measured by Image J.

### 4.7. Stomatal Opening Assay 

4 leaves of Col-0, mutants, and transgenic plants grown for 4 weeks under short sunlight conditions were incubated in stomatal buffer (20 mM KCl, 1 mM CaCl_2_, 2.5 mM MES-KOH, pH 6.15) for 2–3 h in light (450 mol m^−2^ s^−1^). Water or ABA was added to the samples. After 2 h of continuous illumination, the lower epidermises of the leaves were observed under the microscope.

### 4.8. Enzymatic Determination

Col-0, mutant, and transgenic plants growing for 20 days were exposed to 20% PEG6000 to simulate drought stress. Leaves were collected at different times to prepare extracts, and the peroxidase activities (CAT, SOD, POD, APX) and hydrogen peroxide (H_2_O_2_) content were measured under specific wavelengths by colorimetry.

### 4.9. qPCR

The seeds of Col-0, OE *PIA1* (OE-1, OE-2), and *pia1* were grown vertically on 1/2 MS solid medium for 14 days. Then, 15 seedlings were transferred to 1/2 MS liquid medium for 12 h and treated with 3 μM ABA or water for 5 h. Experiments were performed under the instructions of a plant RNA rapid extraction kit (Mei5bio, Beijing, China). Reverse transcription was performed according to the instructions of hiscript III RT Supermix for the qPCR (+ gDNA wiper) kit (Vazyme). Primers are listed in Table S1.

### 4.10. Yeast Two-Hybrid and Luciferase Complementation Assay

Paired plasmids were transformed into the receptive states of Saccharomyces cerevisiae strain AH109 by the PEG/LiAC method [69]. After being coated on SD/-Trp/-Leu solid medium and cultured at 28 °C for 3 days, yeast cells were transferred to the specific selection media for yeast growth assessments. X-Gal staining was performed after 3 days of incubation at 28 °C [70]. The interaction was further verified by luciferase complementation assay (LCA), and the experiment was performed as described [71].

### 4.11. Statistical Analysis

All experiments were performed with three replicates. The data were shown as mean ± SD (standard deviation) and analyzed by using Student’s *t*-test at * *p* ≤ 0.05 and ** *p* ≤ 0.01.

## 5. Conclusions

PIA1, a clade F member of PP2C, plays a negative regulatory role in ABA signaling pathway. Its negative regulatory effect may be achieved by interacting with a variety of key ABA signaling elements to reduce the oxidative stress ability of plants under drought stress and down-regulate the expression of ABA response genes, and thereby reduce plant drought tolerance. 

## Figures and Tables

**Table 1:** Analysis of cis-acting elements in PIA1 promoter sequence.

Name of the Element	Sequence	Function of the Element	Number
ABRE	(C)CACGTG	cis-acting element involved in the abscisic acid responsiveness	2
ARE	AAACCA	cis-acting regulatory element essential for the anaerobic induction	6
TC-rich repeats	GTTTTCTTAC	cis-acting element involved in defense and stress responsiveness	1
MBS	CAACTG	MYB binding site involved in drought inducibility	1
CCAAT-box	CAACGG	MYBHv1 binding site	1
MRE	AACCTAA	MYB binding site involved in light responsiveness	1
TGACG-motif	TGACG	cis-acting regulatory element involved in MeJA responsiveness	2
CGTCA-motif	CGTCA	cis-acting regulatory element involved in MeJA responsiveness	2
Box 4	ATTAAT	part of a conserved DNA module involved in light responsiveness	1
G-Box	CACGTG	cis-acting regulatory element involved in light responsiveness	2
I-box	gGATAAGGTG	part of a light-responsive element	2
TCCC-motif	TCTCCCT	part of a light-responsive element	1
TGA-element	AACGAC	auxin-responsive element	1

## Data Availability

The data that support the findings of this study are available from the corresponding author upon reasonable request.

## Supplementary Materials

The following supporting information can be downloaded at: https://www.mdpi.com/article/10.3390/plants12142716/s1, Figure S1: OE *PIA1* and *pia1* mutant identified by PCR and Western blot; Figure S2: The luciferase activity measurement using a luminometer; Table S1: Primers information.
