# Supplementary material for: Arabidopsis Protein Phosphatase PIA1 Impairs Plant Drought Tolerance by Serving as a Common Negative Regulator in ABA Signaling Pathway
Source: Plants (Basel). 2023 Jul 21;12(14):2716. doi: 10.3390/plants12142716 (PMC10384177; doi:10.3390/plants12142716)
Supplement: Supplementary file 1 [file plants-12-02716-s001.zip › plants-2359137-supplementary.pdf]

Supplementary Materials:

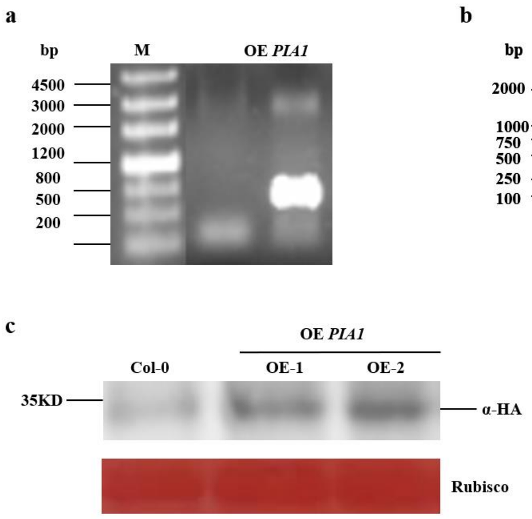

Figure S1. OE *PIA1* and *pia1* mutants identified by PCR and Western blot. **(a)** PCR identification of *PIA1*. **(b)** PCR identification of *pia1* mutant. **(c)** Identification of PIA1 expression.

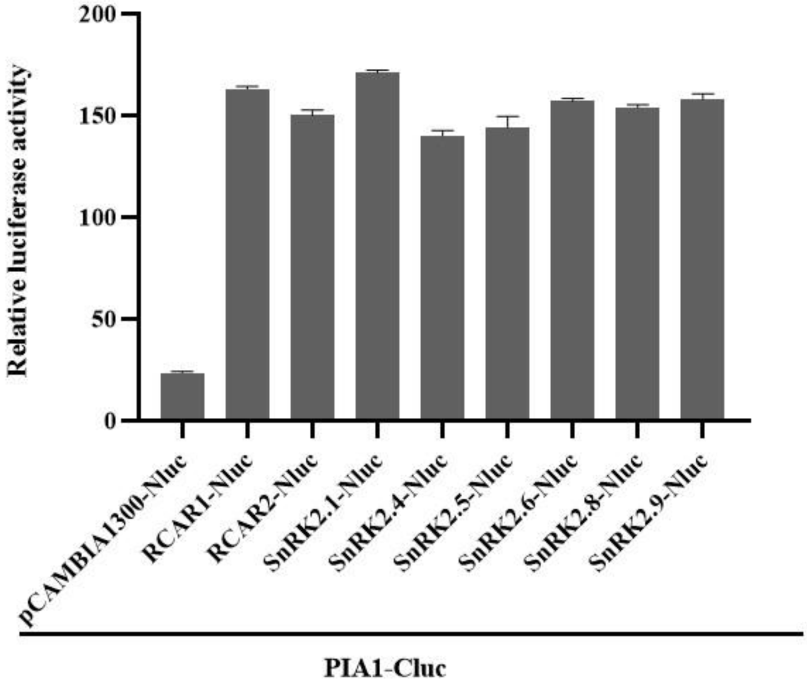

Figure S2. The luciferase activity measurement using a luminometer.

Table S1. Primers information.

Prime name	Primer sequence
pia1	LP：GGCTGCATCAATCAAACAAAC
	RP：CGGTGTTGCTTCTCAACTCTC
	BP：ATTTTGCCGATTTCGGAAC
Actin2	FP：CTTGTTCCAGCCCTCGTTTG
	RP：CAGCGATACCTGAGAACATAGTGG
RD29B	FP：TTCTGACCACACCAAACCCAT
	RP：CAGCCAGTGCCTCATGTCC
ABI3	FP：CTCCGACGTCAAATGTGGCA
	RP：CCACATTCGCCGACGGAGAG
ABI5	FP：CGGGTTTGGATTAGGTTTAG
	RP：GTAGTAGTAGTAGTAATGGACAGA
ABF4	FP：AACAACTTAGGAGGTGGTGGTC
	RP：CTTCAGGAGTTCATCCATGTTC
AD- PIA1	FP：GCCATGGAGGCCAGTGAATTCATGGCAGGCAGAGAGA
AD- PIA1	RP：CAGCTCGAGCTCGATGGATCCTCATCTACAATACCTTTCTGAG
Cluc- PIA1	FP：TACGCGTCCCGGGGCGGTACATGGCAGGCAGAGA
	RP：ACGAAAGCTCTGCAGGTCGACTCATCTACAATACCTTTCT
